# Satellite Tracking and Citizen Science Provide Complementary Insights Into Spatial and Temporal Migration Patterns of Eastern Buzzards

**DOI:** 10.1002/ece3.74701

**Published:** 2026-11-11

**Authors:** Kai Dong, Xu Zhao, Qing Yang, Changhu Lu

**Affiliations:** ^1^ College of Life Sciences Nanjing Forestry University Nanjing Jiangsu China

**Keywords:** citizen science, data integration, eastern buzzard, migration timing, satellite telemetry, stopover sites

## Abstract

Individual bird tracking can resolve continuous migration processes but is limited by small sample sizes, whereas citizen‐science records provide broad spatial and multi‐year coverage but are affected by observation effort and spatial accessibility. However, few studies have evaluated how citizen‐science records and individual tracking correspond across both spatial and temporal components of the same migratory system, including migration routes, stopover sites, migration timing, and broad‐scale spatial coverage. We integrated 105,630 valid satellite locations from 22 rescued and released Eastern Buzzards ( *Buteo japonicus* ), of which 10 completed 27 full migrations, with 25,651 records of this species from eBird and GBIF. We compared the two data sources in terms of migration routes and continental‐scale latitudinal coverage, repeatedly used stopover sites, migration timing, and landscape composition. Both data sources revealed northward migration in spring and southward migration in autumn. The latitudinal spans represented by citizen‐science records were 661 km narrower in spring and 937 km narrower in autumn than those derived from tracking. Five of the 10 repeatedly used stopover sites identified by satellite tracking had citizen‐science records within 100 km during the corresponding migration season. The overlap coefficients between their 24‐h distributions were 0.594 in spring and 0.585 in autumn. Bray–Curtis similarity in landscape composition between the two data sources was 0.545. These results show that the two data sources provide convergent evidence of seasonal migration progression and continental‐scale movements, while local differences highlight potential gaps in citizen‐science coverage.

## Graphical abstract

Using satellite‐tracking data from 22 Eastern Buzzards and citizen‐science records, we examined migration patterns and the relationship between the two data sources. The two sources provided complementary information across spatial and temporal scales.

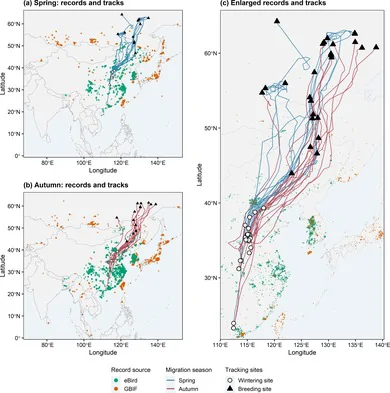

## 1 Introduction

Bird migration is an important life‐history strategy through which birds respond to seasonal changes in resources and environmental conditions (Abrahms et al. 2021). At the individual level, migration involves decisions about departure timing, route choice, diel flight rhythms, and stopover use, which are jointly influenced by internal state, movement and navigation capacity, and the external environment (Nathan et al. 2008; Thorup et al. 2017). The movements of many individuals collectively produce migration waves with distinct timing and direction, as well as seasonal migration corridors and shifts in population distribution (Bauer et al. 2019; Gomez et al. 2025). A comprehensive understanding of bird migration therefore requires both continuous records of individual movements and stopover behavior and population‐level distribution data spanning broad regions and multiple years (Bridge et al. 2011; Kays et al. 2015).

Satellite tracking can directly identify migration timing, routes, flight speeds, and stopover sites and can reveal individual responses to topography, weather, and landscape conditions (Ellis‐Soto et al. 2025). Tracking the same individuals across years can also reveal individual consistency and interannual variation in migration behavior (Franklin et al. 2022). However, equipment and data‐transmission costs constrain sample sizes, while tag lifespan, location frequency, and missing data affect the spatiotemporal resolution of behavioral identification (Lahoz‐Monfort and Magrath 2021). Imbalances in the age, sex, capture location, and population origin of tracked individuals may further reduce sample representativeness (Hebblewhite and Haydon 2010). Satellite tracking can therefore describe the migration of tagged individuals accurately but cannot readily represent long‐term distributional changes in larger populations. In contrast, citizen‐science data have been used to reveal migration timing and seasonal differences, potential routes and stopover areas, and distributional changes across the annual cycle (Supp et al. 2015; Bounas et al. 2020; Fink et al. 2020). Widely used biodiversity data platforms include eBird, which compiles bird observations across regions and years (Sullivan et al. 2009), and GBIF, which aggregates open species‐occurrence records from diverse sources (Amano et al. 2016). Together, these platforms provide broad coverage for population‐level migration studies (Chandler et al. 2017). However, these data do not provide continuous individual trajectories, and their spatiotemporal distributions are influenced by human population density, road accessibility, observation effort, and species detectability (Kelling et al. 2019; Johnston et al. 2023). Data completeness is also lower at fine resolutions and in under‐observed regions such as northeastern Asia (La Sorte and Somveille 2020). Citizen‐science records are therefore well suited to describing broad‐scale, long‐term migration patterns but provide limited information on continuous individual routes, short stopovers, and local movements.

Previous studies have compared or integrated citizen‐science records with individual tracking data, including satellite telemetry and geolocators, to examine different components of bird migration. Studies focusing on stopover use combined geolocator, weather radar, and eBird data to identify a major Tree Swallow stopover area (Laughlin et al. 2013). Geolocator and ringing data were also used to assess seasonal stopover regions predicted from eBird (Heim et al. 2020). Other studies focused on broader‐scale movements. Broad seasonal routes for 12 species were mapped by integrating tracking and band re‐encounter data with eBird records, while eBird distributions and limited tracking data were used to infer migration routes, timing, and connectivity (Meehan et al. 2022; Fuentes et al. 2023). Buderman et al. (2025) combined telemetry and eBird distribution data to model spring migration in Golden Eagles. More recently, Chen et al. (2026) used BirdFlow to model population‐level movements of 153 North American migratory bird species by integrating eBird abundance estimates with GPS tracks, banding recoveries, and radio telemetry data. Few studies have combined citizen‐science records and individual tracking data to examine both broad‐scale migration patterns and repeatedly used stopover sites within the same migratory system. In this study, we integrated satellite‐tracking data from Eastern Buzzards with eBird and GBIF records and compared the spatial and temporal migration patterns represented by the two data sources in four respects: (1) broad‐scale migration patterns and continental‐scale latitudinal coverage; (2) the spatial correspondence between repeatedly used stopover sites identified by satellite tracking and citizen‐science records during the corresponding migration season; (3) spring and autumn migration progression and 24‐h temporal distributions; and (4) landscape composition surrounding records from the two sources and the similarity between them.

## 2 Materials and Methods

### 2.1 Species and Data Sources

The Eastern Buzzard ( *Buteo japonicus* ) is a medium‐sized diurnal raptor widely distributed across East Asia. Northern populations migrate between breeding areas in Northeast Asia and wintering areas farther south, mainly using soaring and gliding flight. Its broad distribution and conspicuous daytime flight make it one of the raptors most readily recorded in citizen‐science observations, particularly during raptor watching (Hijikata et al. 2022; Nakahara et al. 2022).

From March 16, 2019 to March 14, 2024, satellite‐tracking data were obtained from 22 Eastern Buzzards that had been rescued, rehabilitated, assessed as fit for release, and subsequently released in eastern China as part of a long‐term rescue‐and‐release monitoring program. Each bird was fitted with a 17‐g Global Messenger satellite transmitter (HQBG2715S; Hunan Global Messenger Technology Co. Ltd., China), attached using a backpack‐style harness and representing less than 3% of the bird's body mass. The present study involved no additional animal capture, handling, tagging, specimen collection, or transmitter deployment. The original rescue, rehabilitation, release, and satellite‐tracking procedures complied with the Wildlife Protection Law of the People's Republic of China and the Guidelines to the Use of Wild Birds in Research (Fair et al. 2010). Locations were generally recorded at approximately 1‐h intervals. A total of 105,751 raw locations were obtained during the study period. After removing implausible locations, duplicate records, and obvious spatial outliers, 105,630 valid locations remained. Because all tracked birds were released in eastern China, the tracking sample may not represent all migratory populations or routes within the study region. Individual body masses, release sites, and transmitter details, together with information on rescue and rehabilitation, are provided in Table S1.

The spatial extent for compiling citizen‐science records was defined according to the migration range of Eastern Buzzards in East Asia, encompassing eastern and northeastern China, the Russian Far East, and adjacent regions. Public observation records were obtained from the eBird Basic Dataset (EBD_relJun‐2026; eBird 2026) and GBIF (GBIF.org 2026). After standardizing species names and removing records with missing dates or coordinates, implausible locations, or duplicates, we retained 25,651 records dated through December 31, 2025, including 22,059 eBird records and 3592 GBIF records.

### 2.2 Migration Timing and Stopover Sites

A complete migration event was defined as an individual leaving one stable seasonal range, moving continuously in a consistent direction, and entering another stable range. In total, 10 individuals contributed 27 complete migration events, including 13 in spring and 14 in autumn. For the comparison of seasonal latitudinal spans, citizen‐science records were selected within the period covered by tracked migrations in the corresponding season and year and deduplicated by date and coordinates. Seasonal latitudinal spans were calculated separately for daily tracking locations and matched citizen‐science records using the central 90% latitude range (5th–95th percentiles). The latitude difference was converted to kilometers at 111.195 km per degree.

Daily mean latitude and longitude were calculated separately for satellite‐tracking locations and citizen‐science records. Annual trends were fitted using generalized additive models with source‐specific cyclic smooths, weighted by the square root of the number of contributing individuals (tracking) or years (citizen science). Approximate 95% confidence intervals were obtained from the model predictions and their standard errors.

All timestamps used in the diel analysis were converted to Beijing Time (UTC + 8). For the tracking data, we retained consecutive locations separated by 1–2 h and classified movements with speeds of at least 5 km h^−1^ as active migration (Dodge et al. 2014). For the citizen‐science data, the diel analysis was restricted to eBird records with checklist start times. Tracked movements classified as active migration and eBird records were grouped by hour of day, and counts were converted to hourly proportions separately for each season and data source. The overlap between the two 24‐h distributions was quantified using a discrete overlap coefficient (Pastore and Calcagnì 2019):

$$O=\sum_{h=0}^{23}\min\left(T_{h},E_{h}\right)$$

where (*T*_*h*_) and (*E*_*h*_) represent the proportions of tracked movements classified as active migration and eBird records, respectively, occurring during hour (*h*). *O* ranges from 0 to 1, with higher values indicating greater similarity between the two diel distributions.

A stopover event was defined as a continuous period during migration lasting at least 2 days, during which both daily displacement and the maximum activity radius did not exceed 10 km and the net displacement between the first and last locations did not exceed 20 km. Stopover events were assigned to the same spatial cluster when the distance between the centers of any pair of events within the cluster did not exceed 50 km. A spatial cluster used during at least two independent migration events was defined as a repeatedly used stopover site. Citizen‐science records around each site were summarized as the number of independent observation days represented by eBird and GBIF records within 100 km of the site center during the corresponding migration season. The spring period extended from 16 March to 30 June, and the autumn period from 30 August to 9 January of the following year. Multiple records from the same site on the same date were counted as a single observation day.

### 2.3 Landscape Composition

We used the 2021 MCD12Q1 Collection 6.1 land‐cover product (IGBP classification; 500‐m resolution) to characterize the landscape composition surrounding each record unit (Friedl and Sulla‐Menashe 2022). Land‐cover classes were combined into nine categories: forest, shrubland, savanna, grassland, wetland, cropland, urban, barren, and water. Using the center of the 500‐m pixel containing each record as the center point, we calculated the area proportion of each landscape category within a 20‐km neighborhood. To reduce the disproportionate influence of high‐frequency tracking locations and clustered observations, tracking data from the migration periods were aggregated by individual, date, and 500‐m pixel. All citizen‐science records within the standardized migration‐season windows were retained. We calculated the mean proportion of each landscape category separately for the two data sources and used Bray–Curtis similarity to quantify the similarity in their nine‐category landscape composition (Bray and Curtis 1957). All analyzes were conducted in R 4.2.3 and ArcGIS Desktop 10.8.

## 3 Results

### 3.1 Migration Routes

Satellite‐tracking data documented 27 complete migrations by 10 Eastern Buzzards, including 13 in spring and 14 in autumn. The tracked movements formed a broad northeast–southwest corridor through eastern and northeastern China and extended into the Russian Far East and adjacent regions, although routes and seasonal endpoints varied among migration events (Figure 1).

**FIGURE 1:**
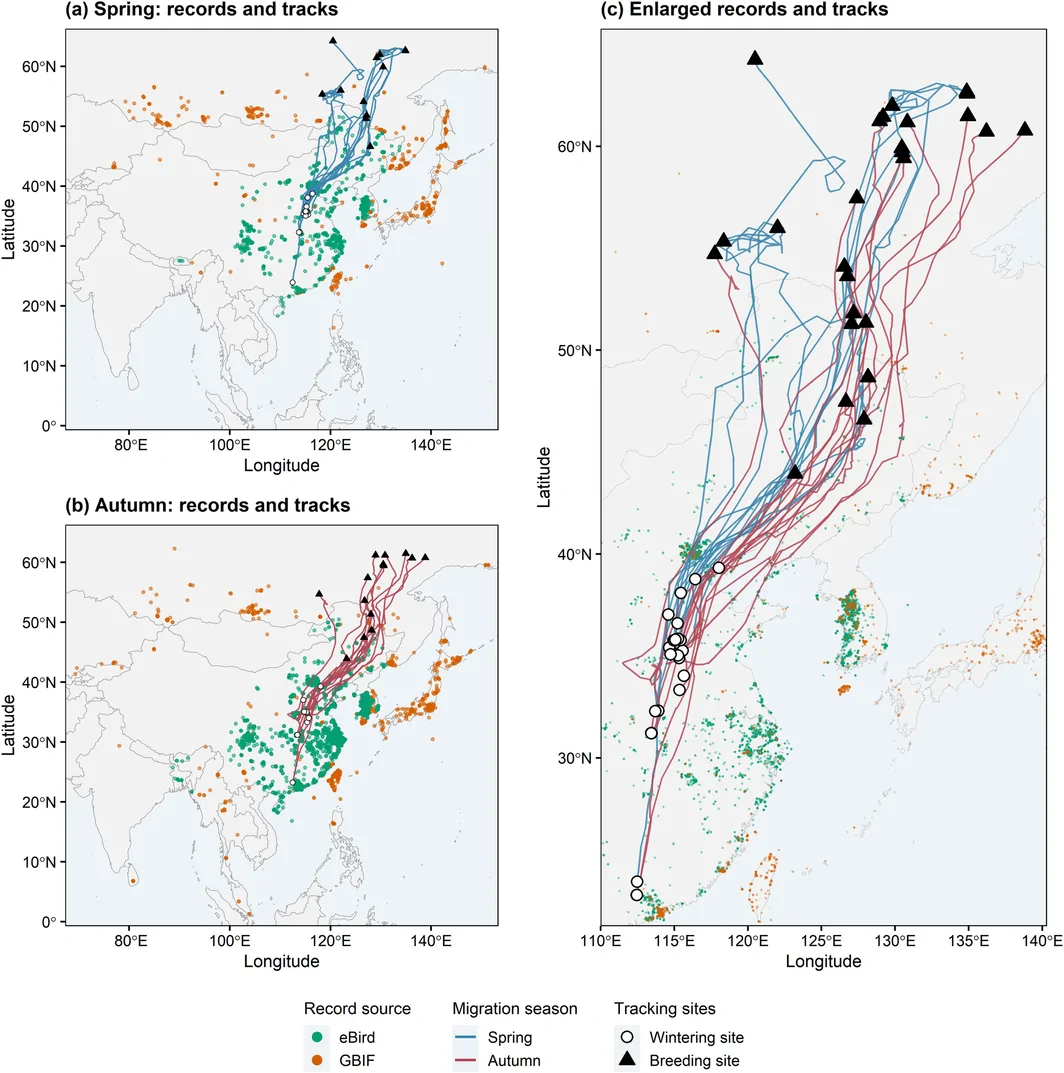
Spring and autumn citizen‐science records, migration routes and seasonal endpoints of Eastern Buzzards. Panels (a) and (b) show spring and autumn records and routes, respectively; panel (c) shows an enlarged view combining both seasons. Citizen‐science records are from eBird and GBIF, and tracking routes represent 27 complete migration events (13 in spring and 14 in autumn) by 10 individuals wintering in eastern China.

Both satellite‐tracking and citizen‐science records showed seasonal latitudinal spans exceeding 2000 km. In spring, the 5th–95th percentile spans were 3059 km for satellite tracking and 2398 km for citizen science; in autumn, the corresponding spans were 2957 and 2020 km. The latitudinal span represented by citizen‐science records was therefore 661 km narrower in spring and 937 km narrower in autumn than that represented by satellite tracking, with a larger difference in autumn (Figure 2).

**FIGURE 2:**
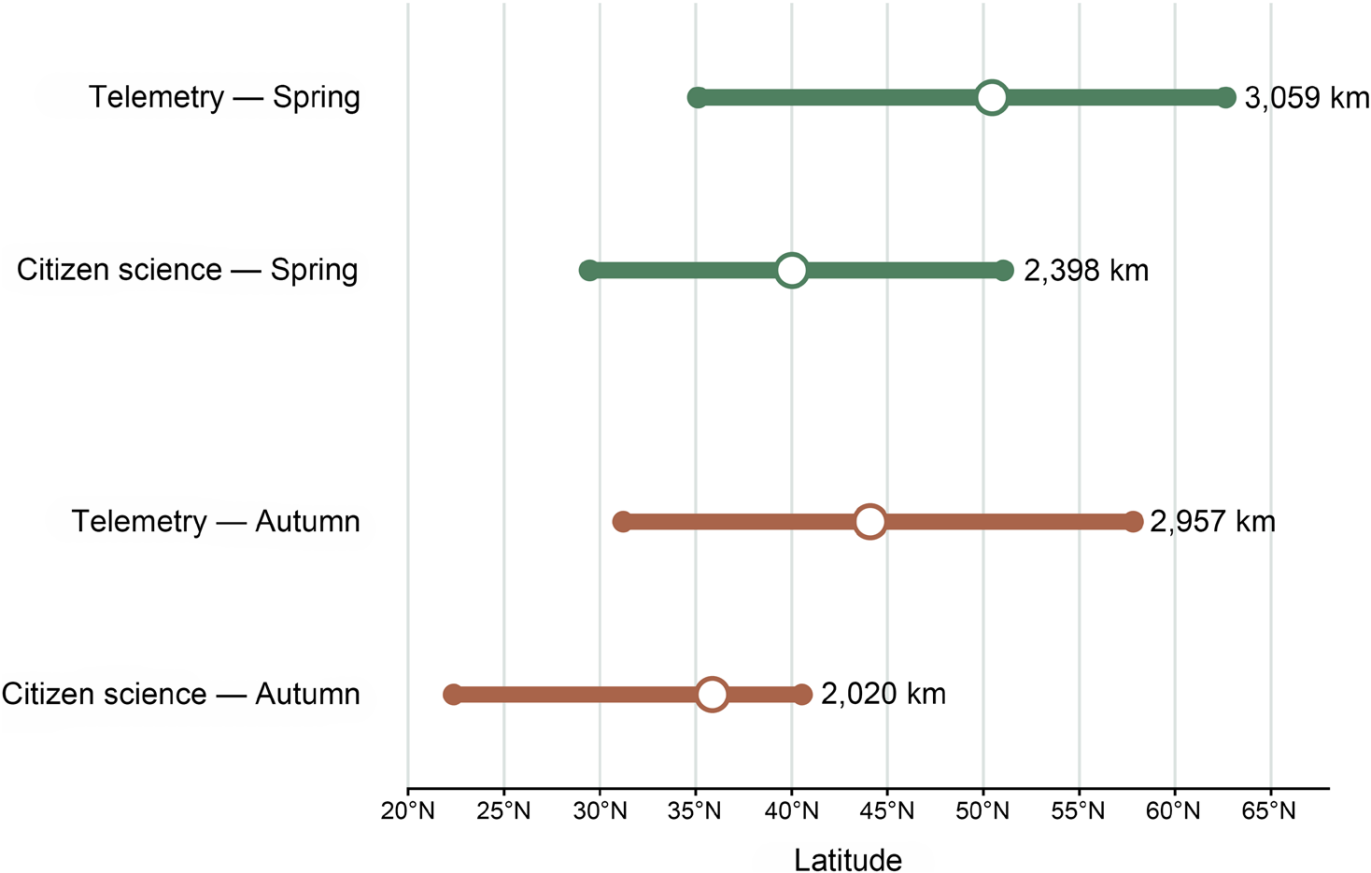
Seasonal latitudinal coverage represented by satellite‐tracking and citizen‐science records. Horizontal lines indicate the latitude ranges containing the central 90% of records (5th–95th percentiles) for the corresponding data source and migration season. Open circles indicate median latitudes, and labels show the corresponding latitudinal spans in kilometers.

### 3.2 Migration Stopover Sites

Clustering identified 10 repeatedly used stopover sites spanning 34.4°–52.7° N. Each site contained 2–5 stopover events, and mean stopover duration ranged from 2 to 10 days among sites. Site R6 contained five stopover events representing three migrations by two individuals. Of the 10 sites, R1, R2, R3, R5, and R7 had citizen‐science records within 100 km, with 22, 13, 2, 15, and 13 independent observation days, respectively (Figure 3). No citizen‐science records were found within 100 km of the remaining five sites during the corresponding migration season.

**FIGURE 3:**
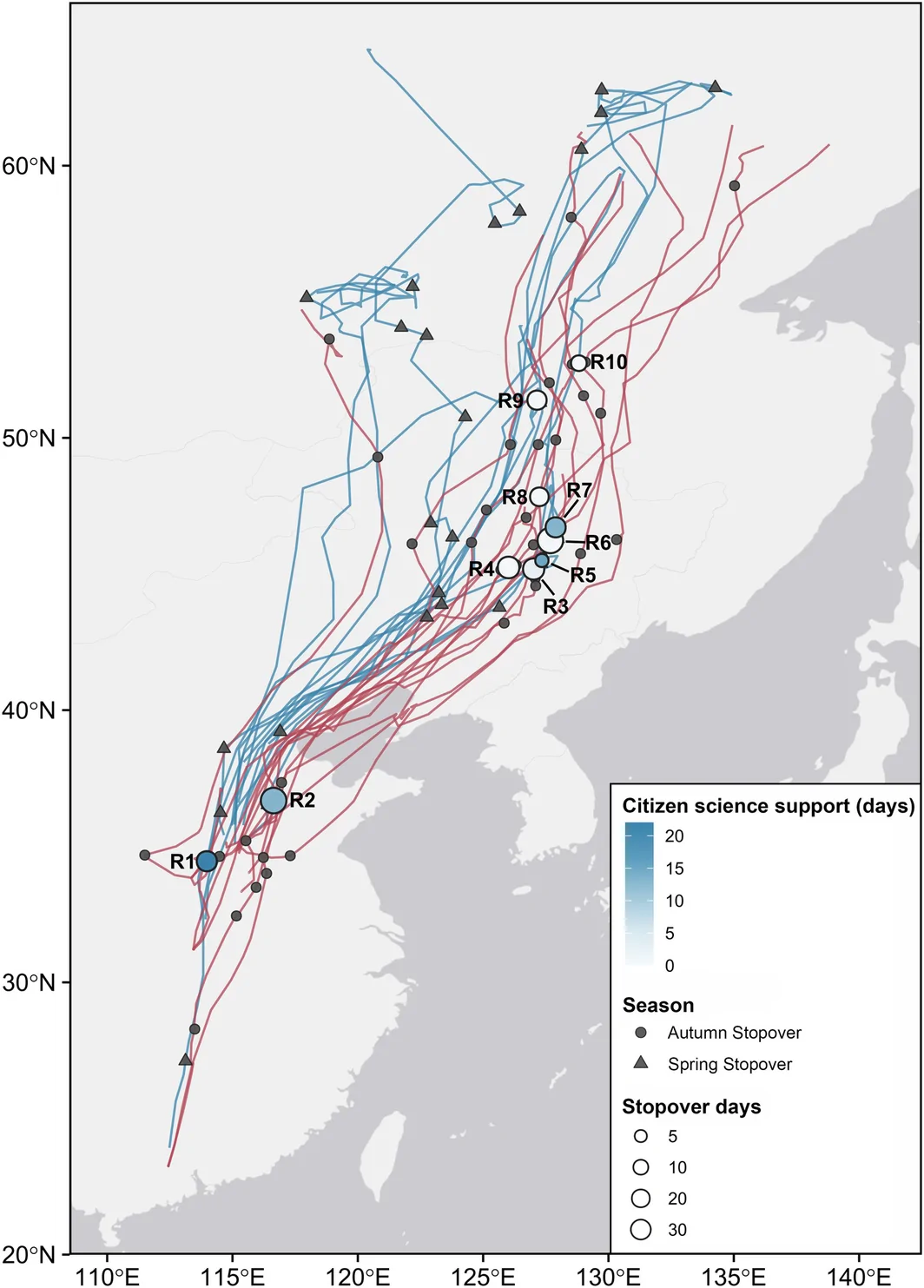
Repeatedly used stopover sites of Eastern Buzzards and nearby citizen‐science records.

### 3.3 Migration Timing

The daily mean latitudes of satellite‐tracking locations and citizen‐science records showed similar seasonal changes. From April to June, the smoothed daily mean latitude increased from approximately 34° N to 53° N for satellite tracking and from 31° N to 49° N for citizen science, reflecting northward spring migration. From September to November, it declined from approximately 55° N to 35° N for satellite tracking and from 50° N to 29° N for citizen science, reflecting southward autumn migration. By contrast, daily mean longitude showed markedly different patterns between the two data sources. The mean longitude of tracking locations increased rapidly from April onward and remained high throughout summer, whereas that of citizen‐science records decreased over the same period and increased again from autumn onward. Overall, the two data sources showed similar seasonal latitudinal patterns but weaker correspondence in longitude (Figure 4).

**FIGURE 4:**
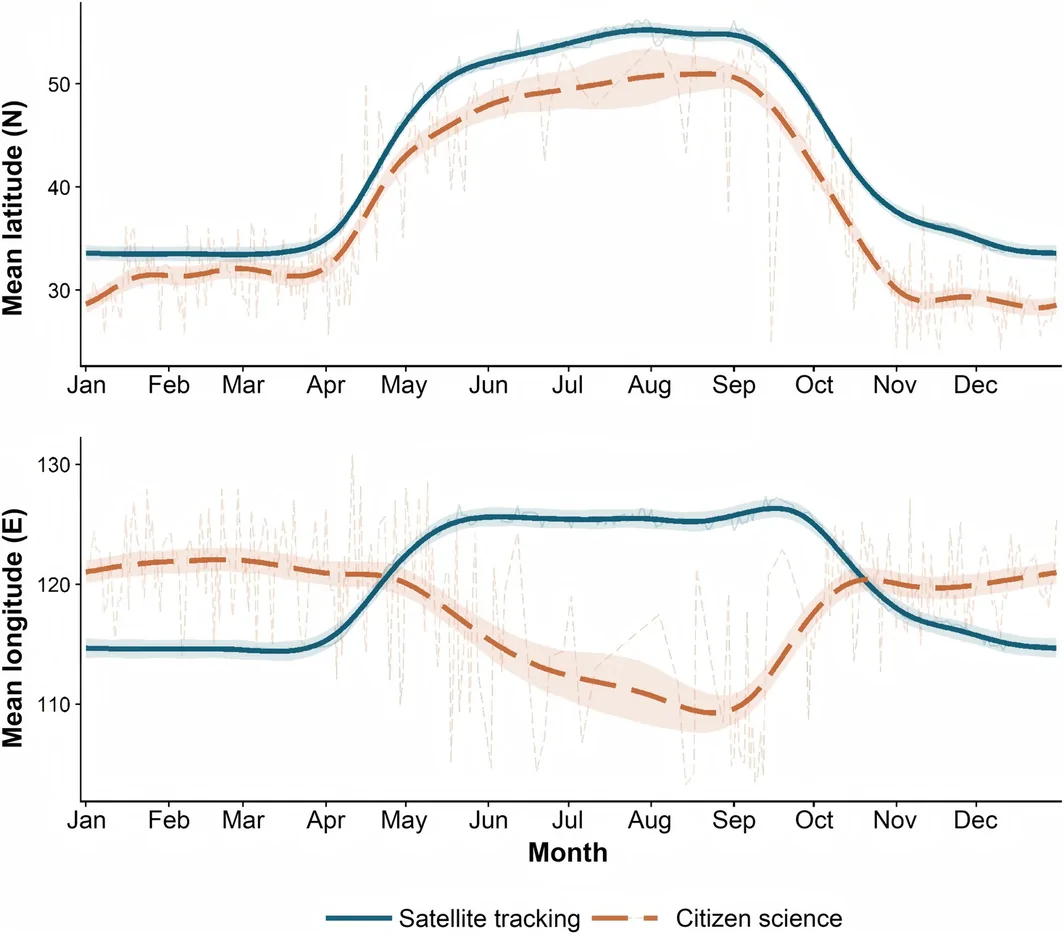
Seasonal changes in mean latitude and longitude from tracking and citizen‐science data. Thin lines show daily means, whereas thick lines and shaded bands represent smoothed trends and their 95% confidence intervals, respectively.

Tracked active‐migration movements and eBird records with checklist start times were both concentrated during daylight hours. eBird records peaked at 08:00 in both spring and autumn, whereas tracked migration activity peaked at 10:00 in spring and 12:00 in autumn and remained high around midday (Figure 5).

**FIGURE 5:**
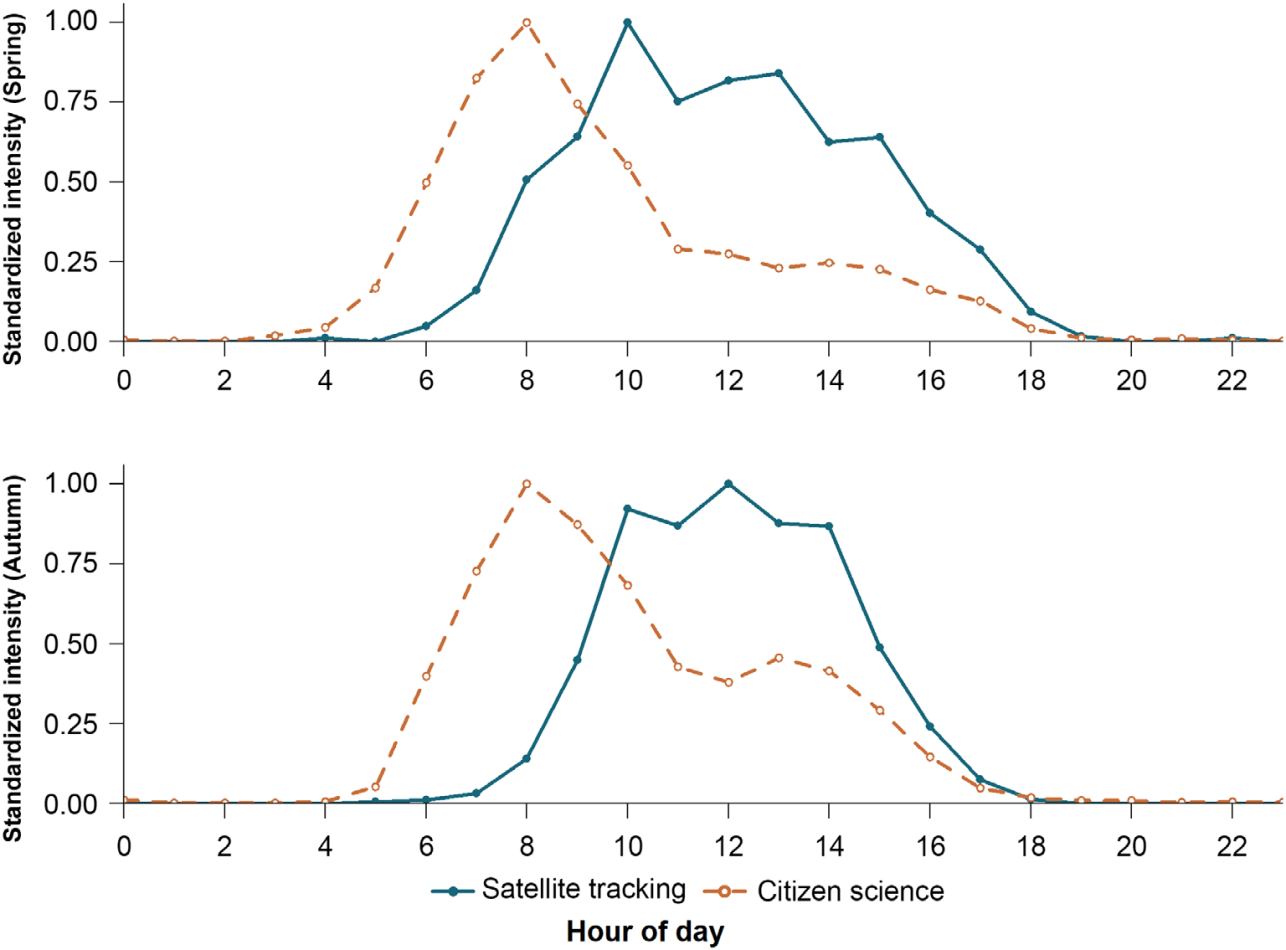
Standardized hourly distributions of tracked migration activity and eBird records during spring and autumn migration.

The overlap coefficient (O; ranging from 0 for no overlap to 1 for complete overlap) between the hourly distributions of tracked migration activity and eBird records was 0.594 in spring and 0.585 in autumn. Overlap was similar between seasons, with a slightly higher value in spring than in autumn.

### 3.4 Landscape Composition During Migration

The landscapes surrounding satellite‐tracking units during migration were dominated by savanna (40.9%) and cropland (35.0%). In contrast, those surrounding citizen‐science records were dominated by urban land (25.2%), water (21.1%), and cropland (17.7%). The main differences between the two data sources occurred in savanna, urban land, and water. Bray–Curtis similarity across the nine landscape categories was 0.545, indicating partial similarity in overall landscape composition between the two data sources (Figure 6).

**FIGURE 6:**
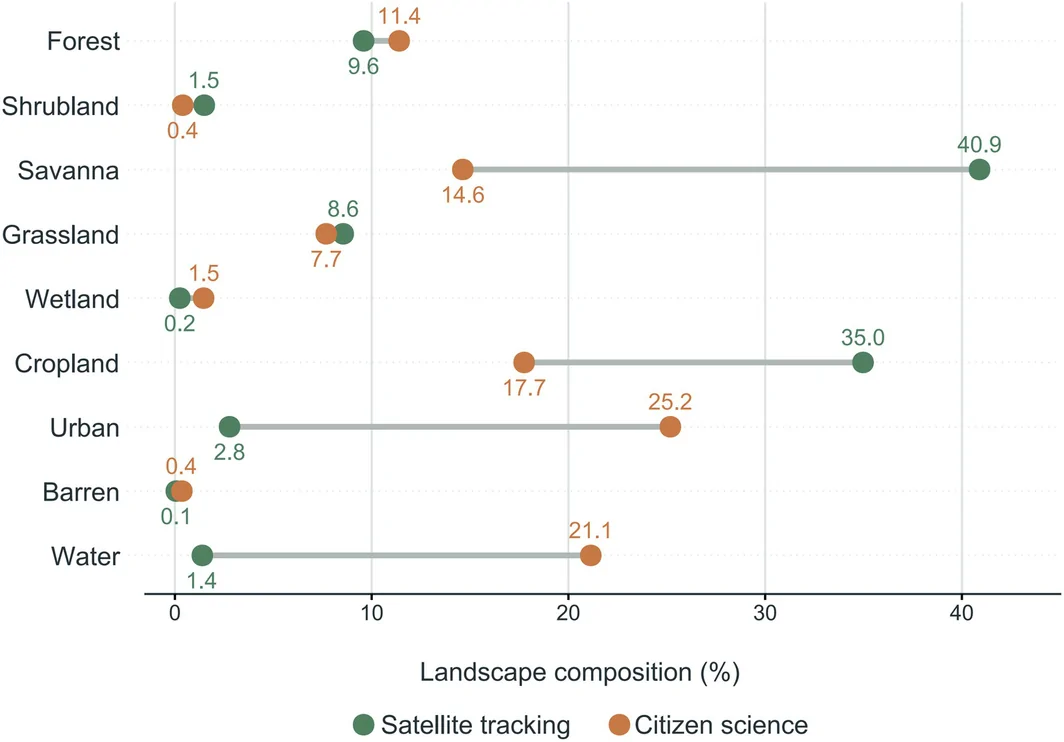
Landscape composition in 20‐km neighborhoods around satellite‐tracking and citizen‐science units during migration.

## 4 Discussion

### 4.1 Migration Routes and Continental‐Scale Coverage

Both data sources showed latitudinal coverage exceeding 2000 km, but the citizen‐science spans were 661 km narrower in spring and 937 km narrower in autumn than those derived from tracking. Seasonal changes in latitude were broadly similar between the two data sources, with both showing northward migration in spring and southward migration in autumn, whereas changes in mean longitude differed markedly. This suggests that agreement in the overall migration direction does not necessarily imply identical spatial patterns. Previous tracking studies have identified at least four major routes: an eastern China–Northeast China–Russian Far East continental route; an alternative autumn route through the Changbai Mountains, Liaodong Peninsula, and Bohai Sea; a Tsushima Strait–Korean Peninsula–eastern Eurasian margin route; and a Japanese Archipelago–Hokkaido or Sakhalin route (Hijikata et al. 2022; Nakahara et al. 2022; Yang and Lu 2026). Citizen‐science records may include individuals from different geographic populations and migration corridors, whereas the tracked birds were all released in eastern China and mainly represented birds using a relatively concentrated continental corridor. The longitudinal differences may reflect variation in population origins and route composition, together with uneven observation coverage and effort.

### 4.2 Stopover Sites and Monitoring Gaps

Of the 10 repeatedly used stopover sites identified by satellite tracking, five had citizen‐science records within 100 km during the corresponding migration season. Continuous individual trajectories allowed satellite tracking to identify the locations and durations of stopover events at relatively fine spatial and behavioral resolution, whereas citizen‐science records provided discontinuous occurrence records and could indicate regional occurrence around these sites. Citizen‐science coverage was also incomplete in remote regions, where opportunistic records are constrained by human population density, road accessibility, and observation effort. Sites in lower‐latitude eastern China generally had more independent observation days, whereas fewer were recorded around sites in Northeast China and the Russian Far East, consistent with known spatial biases in opportunistic biodiversity records (Mair and Ruete 2016; Johnston et al. 2021). Thus, satellite tracking was better suited to delineating repeatedly used stopover sites, whereas citizen‐science records provided additional records of Eastern Buzzards around these sites where observation coverage was sufficient.

### 4.3 Migration Timing Across Scales

At the seasonal scale, both data sources showed northward migration in spring and southward migration in autumn. Heim et al. (2020) found that eBird‐based distribution predictions agreed more closely with tracking records in winter than during spring and autumn migration. Tracking describes the seasonal movements of individual birds, while citizen‐science records show where a species is recorded in different seasons (Fuentes et al. 2023).

eBird records peaked at 08:00 in both spring and autumn, whereas active migration by tracked individuals peaked at 10:00 in spring and 12:00 in autumn. These differences may reflect both observation processes and migration behavior. Citizen‐science records are influenced by observation time, observer effort, and species detectability (Johnston et al. 2023). Birdwatching activity also tends to be concentrated in the early morning (Frątczak et al. 2020), which may partly explain the eBird peak at 08:00. In contrast, the migration activity of soaring birds is influenced by updrafts and wind conditions, potentially causing the peaks identified by tracking to occur later (Alioto et al. 2025). Although the peak hours differed, eBird checklist start times overlapped with the daytime periods of active migration recorded by tracking. Tracking therefore provides a reference for interpreting the hourly distribution of citizen‐science records by identifying when tagged birds are actively migrating.

### 4.4 Landscape Composition and Sampling Bias

Bray–Curtis similarity across the nine landscape categories was 0.545, indicating partial correspondence in landscape composition between the two data sources. Landscapes surrounding satellite‐tracking units were dominated by savanna and cropland, whereas urban land and water were more prevalent around citizen‐science records. These differences may partly reflect the observation process underlying citizen‐science data. Birdwatching activity is often concentrated around wetlands and water bodies (Prenda et al. 2024) and in densely populated or accessible areas (Mair and Ruete 2016), while eBird records may disproportionately represent urban environments (La Sorte et al. 2024). The observed landscape differences should therefore be interpreted as the combined outcome of actual bird occurrence, regional landscape availability, and source‐specific sampling bias rather than as direct differences in habitat preference.

### 4.5 Limitations and Future Research

This study was based on 27 complete migrations from 10 rescued and released individuals, including repeated migrations by the same birds, which may limit population‐level inference. eBird and GBIF records are influenced by observation effort, accessibility, and detectability. These factors were not explicitly modeled, so spatial and landscape differences may partly reflect where and how intensively birds were observed, alongside differences in the geographic origins and routes of the birds represented by each source. For eBird checklists, the recorded start time may precede the actual buzzard sighting, and a sighting does not necessarily indicate active migration. The datasets covered different periods, so year‐to‐year variation in migration timing and observation coverage may have influenced the comparison of seasonal patterns. Future studies could expand tracking to populations from different geographic regions and incorporate weather and other environmental data, together with information on observation effort, to further improve comparisons between tracking and citizen‐science records.

## 5 Conclusion

Satellite tracking and citizen‐science records consistently revealed the seasonal migration directions of Eastern Buzzards. Five of the 10 repeatedly used stopover sites identified by satellite tracking had citizen‐science records within 100 km during the corresponding migration season. Together, the two data sources provide complementary information on individual movements and seasonal occurrence, while their differences highlight potential gaps in citizen‐science coverage.

## Author Contributions

**Kai Dong:** conceptualization (equal), data curation (equal), formal analysis (equal). **Xu Zhao:** data curation (equal), investigation (equal). **Qing Yang:** data curation (equal), investigation (equal). **Changhu Lu:** conceptualization (equal), funding acquisition (lead).

## Funding

This work was supported by the Priority Academic Program Development of Jiangsu Higher Education Institutions (PAPD 2022–2025).

## Conflicts of Interest

The authors declare no conflicts of interest.

## Supporting information


**Table S1:** Individual information and transmitter specifications for the 22 satellite‐tracked Eastern Buzzards.

## Data Availability

The processed data and R code required to reproduce the analyzes and figures are available in a Zenodo record. The original eBird and GBIF occurrence records can be obtained from the sources cited in the manuscript. Individual information for the tracked birds is provided in Table S1.
